# Reporting of feasibility factors in publications on integrated treatment programs for women with substance abuse issues and their children: a systematic review and analysis

**DOI:** 10.1186/1478-4505-10-37

**Published:** 2012-12-07

**Authors:** Joanna Henderson, Karen Milligan, Alison Niccols, Lehana Thabane, Wendy Sword, Ainsley Smith, Susan Rosenkranz

**Affiliations:** 1 Child, Youth and Family Program, Centre for Addiction and Mental Health, Department of Psychiatry, University of Toronto, 80 Workman Way, Toronto, ON, M6J 1H4, Canada; 2 Department of Psychology, Ryerson University, 350 Victoria Street, Toronto, ON, M5B 2K3, Canada; 3 Department of Psychiatry and Behavioural Neurosciences, McMaster University, McMaster Children's Hospital-Chedoke Site, Holbrook Building, Hamilton, ON, L8N 3Z5, Canada; 4 Department of Clinical Epidemiology and Biostatistics, McMaster University, St. Joseph's Healthcare, Biostatistics/FORSC, 50 Charlton Avenue East, Hamilton, ON, L8N 4A6, Canada; 5 School of Nursing, McMaster University, Hamilton, ON, L8N 3Z5, Canada; 6 Department of Psychology, Child, Youth and Family Program, Centre for Addiction and Mental Health, 80 Workman Way, Toronto, ON, M6J 1H4, Canada

**Keywords:** Knowledge translation, Feasibility, Substance abuse, Women, Treatment, Review

## Abstract

**Background:**

Implementation of evidence-based practices in real-world settings is a complex process impacted by many factors, including intervention, dissemination, service provider, and organizational characteristics. Efforts to improve knowledge translation have resulted in greater attention to these factors. Researcher attention to the applicability of findings to applied settings also has increased. Much less attention, however, has been paid to intervention feasibility, an issue important to applied settings.

**Methods:**

In a systematic review of 121documents regarding integrated treatment programs for women with substance abuse issues and their children, we examined the presence of feasibility-related information. Specifically, we analysed study descriptions for information regarding feasibility factors in six domains (intervention, practitioner, client, service delivery, organizational, and service system).

**Results:**

On average, fewer than half of the 25 feasibility details assessed were included in the documents. Most documents included some information describing the participating clients, the services offered as part of the intervention, the location of services, and the expected length of stay or number of sessions. Only approximately half of the documents included specific information about the treatment model. Few documents indicated whether the intervention was manualized or whether the intervention was preceded by a standardized screening or assessment process. Very few provided information about the core intervention features versus the features open to local adaptation, or the staff experience or training required to deliver the intervention.

**Conclusions:**

As has been found in reviews of intervention studies in other fields, our findings revealed that most documents provide some client and intervention information, but few documents provided sufficient information to fully evaluate feasibility. We consider possible explanations for the paucity of feasibility information and provide suggestions for better reporting to promote diffusion of evidence-based practices.

## Background

In the past decade, there has been a substantial increase in attention to the knowledge translation and exchange process in substance abuse and mental health treatment. The emerging field of translational research has provided information on facilitators and barriers to implementation of empirically-based practices in applied settings [1]. It is now understood that the diffusion of new clinical practices is a complex process impacted by many factors, including characteristics of the new practice, the dissemination process, the clinician or decision-maker, the organization, and the broader socio-cultural context [2-7] (for a review see Greenhalgh et al., 2004 [8]). For example, adoption and implementation of a new intervention or treatment innovation has been found to occur more frequently when the innovation is perceived as having advantages over existing practices [5,8,9], is perceived as being compatible with existing values and practices [3,5,8,10], is documented in such a way that is easy understand and use [5,7,8], and the risks of implementation are low [8]. In addition, the likelihood of diffusion success is improved if the innovation can be adapted to meet local needs [5,8,11].

At the organizational level, attributes that affect the adoption and implementation of innovations include the availability of the necessary staff, financial and other resources [5,7,8,12], and agency size and culture [5,7-10,12]. In addition, in situations where programs are collaborative in nature and require the coordinated efforts of two or more organizations, successful program implementation is strongly affected by the organizations’ level of commitment to, and engagement in, the collaboration [5,13,14]. Additional factors that have been suggested as important in the adoption and implementation of new intervention approaches by community-based service providers include the type and amount of training and supervision required to implement the treatment [3,7,8,15], the relevance of the studied client group and clinical setting to the potential adopter’s clients and clinical setting [16-18], the availability of ongoing consultation around both the clinical issues and the implementation issues [6,9,12], and potential implementation obstacles and solutions [9,15,19].

These factors have been brought together into conceptual models that try to capture the complexity of the process by articulating the phases of implementation and the factors that are important at each phase (see [8] and [12]). Moreover, recent discussions have highlighted the need for a shift from traditional linear models of intervention development and translation to bi-directional and systems models that attend to the clinical, staff, organizational and system contexts of service delivery from the outset of intervention development and evaluation through to long term maintenance of the new intervention [1-3,8,12,16,20,21].

In the context of heightened awareness about the factors impacting the diffusion process, translational research has expanded to include empirical evaluations of specific interventions to improve diffusion success (e.g., clinical practice guidelines) and increased discussion regarding new approaches to conducting and reporting on research in order to facilitate the movement of research to practice in real world settings. Conceptual frameworks, such as PARiHS (Promoting Action on Research Implementation in Health Services; [21]) and RE-AIM (reach, effectiveness, adoption, implementation, and maintenance; [2]) have been developed to capture the complexity of the knowledge translation process, to aid in the planning, conduct and evaluation of intervention research and transportability efforts, and to improve translation success. Notably, these frameworks emphasize the importance of ensuring that information relevant to treatment efficacy in academic and real-world settings is available to service providers and decision-makers to help them to answer the question “Will this treatment be efficacious in my context?”

Another important domain in understanding the research to practice gap, however, is the issue of *feasibility* or the extent to which an intervention can be implemented successfully in a specific context [22]. Whereas tools such as RE-AIM can provide critical assistance to service providers and decision-makers in evaluating the generalizability and relevance of evidence (“Will this program *work* in my setting, with my patients, under our conditions?” (*italics added;*[2]), little is available to aid service providers and decision-makers in answering another important question: “Is this program *feasible* in my setting, with my patients, under our conditions?” This is despite indications that innovations that are perceived as more feasible are more likely to be adopted [8] and calls for greater attention to the role of feasibility in the implementation process [23]. This gap, in part, may reflect a continued over-representation of approaches that emphasize helping treatment developers and researchers move service providers and decision-makers more effectively toward research utilization and a continued under-representation of approaches that integrate and reflect the unique perspectives of the target audience (i.e., service providers and decision-makers).

Rogers’ [5] Diffusion of Innovation model was developed to provide a translation model framed from an audience perspective [1], allowing for consideration of feasibility. The model delineates the effects of service provider (e.g., self-efficacy, concern), treatment innovation (e.g., compatibility, complexity, relative advantage), organizational (e.g., culture, size, resources) and dissemination (e.g., type of approach, availability of interpersonal support) variables on diffusion success. Feasibility and perceived feasibility have as their primary theoretical underpinning the notion of compatibility or perceived fit [22]. Indeed, the extent to which an innovation fits with or matches the existing values, tasks, and duties of an organization and its individuals has been argued as a key determinant of implementation success [8,12,24]. In addition to compatibility, perceptions of fit and feasibility include consideration of the suitability of the innovation for the particular provider, setting, and client group (sometimes referred to as “appropriateness” [22]), the resources required to successfully implement the innovation [23], and the innovation’s perceived or actual utility [22]. Moreover, Schoenwald and Hoagwood [24] have suggested that successful adoption and implementation of new practices will depend on the extent to which practitioners perceive the innovation to be similar or different from existing practices in six domains: the intervention itself; the practitioners delivering the intervention; the clients receiving the intervention; the service delivery context; the organizational context; and the service system context. Accordingly, in the current review, we used Rogers’ Diffusion of Innovation model as a framework, and integrated the dimensions identified by Schoenwald and Hoagwood [24] as important. Building upon these works, we propose a set of feasibility criteria based on factors that have been shown to increase the likelihood of adoption and implementation of new practices by service providers. In addition, we examined the extent to which these criteria were met in the intervention literature in one specific area: integrated treatment programs for women with substance abuse issues and their children (programs that include on-site pregnancy-, parenting-, or child-related services with addiction services).

### Interventions for women with substance use issues who are pregnant or parenting

Integrated treatment programs for women with substance abuse issues and their children were developed out of an awareness of the complex and unique needs of this population. Although rates of substance use generally are lower for women than for men [25] the physical and mental health consequences and correlates can be more profound for women [26]. Given that the majority of women with substance abuse problems are of child-bearing age [27], many women facing these challenges are pregnant or parenting. Indeed, maternal substance abuse has been associated with parenting capacity risks and an increased likelihood that children are exposed to maltreatment and neglect [28].

The unique challenges of women with substance abuse issues began receiving increased attention in the late 1980s and early 1990s resulting in federal initiatives in the U.S. to provide comprehensive services to women with substance abuse issues who were pregnant and/or parenting. Under various initiatives beginning in the early 1990s through the Substance Abuse and Mental Health Services Administration (SAMHSA), the Centers for Substance Abuse Prevention (CSAP) and Treatment (CSAT), and the National Institute on Drug Abuse (NIDA), over 100 projects were funded to develop and evaluate comprehensive women-specific treatment services that integrated substance abuse and pregnancy and/or parenting-related services. One of the purposes of these initiatives was to develop model programs that could be replicated and to gather data across demonstration project sites [29,30]. Many of these projects resulted in publications documenting their implementation and evaluation efforts.

We conducted the present study as part of a systematic review and meta-analysis examining the effects of these and other integrated treatment programs on maternal and child outcomes [31]. While previous publications on this project document the strength of evidence for the positive outcomes of integrated models of service delivery using traditional effect size criteria [32-35], the present study focuses on feasibility criteria and the reporting of these criteria in this body of literature.

## Methods

### Literature search

We used three main strategies to identify outcome studies of intervention programs for women with substance use issues and their children: online bibliographic database searches, checking printed sources, and online searches for grey literature and researchers [36]. First, we searched relevant bibliographic databases (PsycINFO, MedLine, PubMed, Web of Science, EMBASE, Proquest Dissertations, Sociological Abstracts, and CINAHL) for studies published in English from 1990 to 2009, using a subject heading and keyword search for the terms “substance use/abuse, addiction, alcoholism, intervention, treatment, therapeutic, rehabilitation, women, child, mother, infant, mental health, parenting, prenatal” singly and in combination. Five hundred and fifteen potentially relevant records were identified through this process.

Secondly, we manually searched relevant journals in the area (Addiction, Addictive Behaviors, International Journal of the Addictions, Journal of Drug Issues, Journal of Psychoactive Drugs, Journal of Substance Abuse, Journal of Substance Abuse Treatment, Journal of Substance Use, and Substance Use and Misuse) published from 1990 to 2009. Documents that appeared to be relevant on the basis of titles or abstracts were retrieved. Also, we examined reference lists of retrieved articles for potentially relevant documents (no date restrictions).

**Figure 1:**
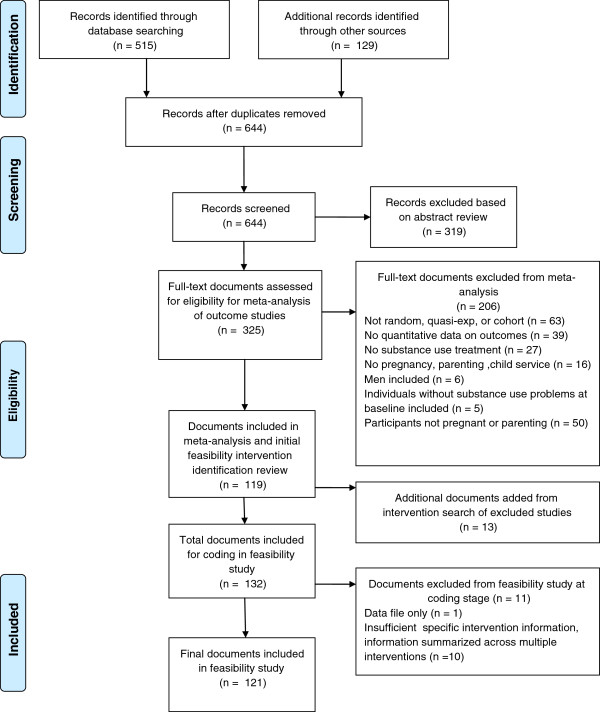
Identification of articles for review.

Finally, we searched on the web using Google for grey literature (e.g., technical reports, program evaluations and summaries, unpublished data; no date restrictions). All researchers identified through these searches, as well as researchers presenting at relevant conferences identified using Google and Cross Currents (Upcoming Events), were contacted by email to request any relevant published or unpublished data. Of the 200 researchers identified and emailed, 48% responded and 28 additional studies were identified. These strategies, together with the manual and reference list searches, resulted in 129 additional records for review, for a total of 644 records. After excluding 319 records based on abstract reviews for relevance 325 documents were retrieved and coded for eligibility for inclusion in the meta-analysis of outcome studies (see Figure 1).

### Eligibility criteria and study inclusion

Eligibility criteria were based on our working definition of integrated programs being substance abuse treatment programs that provide comprehensive services that address substance abuse as well as maternal and child well-being through prenatal services, parenting programs, child care, and/or other child-centred services in a centralized setting. Our criteria were purposefully broad in order to include as many potentially relevant studies with outcome data as possible. Therefore, we included studies in our larger systematic review and meta-analysis if all of the following criteria were met:

1) all participants were women who were pregnant or parenting;

2) all participants had substance abuse problems. We included any study that reported that the participants had a diagnosis of chemical abuse and dependence. However, given that diagnostic or standardized measures of substance use were not routinely used at intake, enrollment in substance abuse treatment was considered to reflect substance abuse at a level that was impacting on daily functioning. Therefore, we included studies where all participants had substance abuse issues that were being addressed by specific substance abuse treatment for abuse of any drug (e.g., cocaine, crack, heroin, marijuana) or alcohol. We excluded any study that included only non-users or those at risk for substance use, and we contacted authors if the issue was unclear or not reported.

3) the treatment program included at least one specific substance use treatment (e.g., individual or group therapy, methadone) and at least one pregnancy, parenting or child (< 16 years) treatment service (e.g., prenatal care, child care, parenting classes);

4) the program was not for men or for women who were not pregnant or parenting;

5) the study design was randomized, quasi-experimental, or cohort; and

6) there were quantitative data on child outcomes or mother outcomes (length of stay, treatment completion, maternal substance use, maternal well-being, or parenting).

Using these criteria, we excluded 206 documents of the 325 retrieved documents (see Figure 1) and considered 119 documents eligible for inclusion in the larger systematic review and meta-analysis. Based on a random sample of 20% of the studies, inter-rater reliability for eligibility coding was high, Kappa = 0.81. We resolved discrepancies by consensus. We estimated the completeness of the search using the capture re-capture method [37]. Based on this method, the estimated number of missing articles is eight (95% confidence interval [CI]: 2, 24), which suggests a 90% capture rate (i.e., the identified studies cover 90% of the search horizon). This reasonably high capture rate suggests that we retrieved a sufficient number of studies to avoid bias in the results of the systematic review.

Given the possibility that some documents excluded through the meta-analysis review process could contain information relevant to feasibility, the 119 documents included in the meta-analysis were reviewed to identify the interventions on which they focused. Then, the documents that had been excluded from the meta-analysis based on study characteristics (i.e., not random, quasi-experimental or cohort; did not provide quantitative outcome data) were reviewed for information about these interventions. Through this process, an additional 13 documents (e.g., reports, qualitative studies) with information about the interventions of interest were identified and included in the feasibility study, for a total sample of 132 documents.

### Coding

We developed a codebook based on factors identified as important in successful innovation diffusion from a review of the diffusion of innovation literature, and theoretical knowledge translation and exchange models. The codebook was pilot tested by project staff and investigators, and revised during early coding. Items were added or deleted, and decisions and clarification of specific items were recorded in a coding policy manual.

#### Document characteristics

The following information was recorded about each document: document type (journal article, book chapter, dissertation, report, other); intervention identification (treatment name and/or location and/or principal investigators); intervention foci (pregnancy; parenting; both); date of publication; document length (in pages); study type (intervention evaluation; moderator study; implementation study; intervention description); study design (randomized/experimental; quasi-experimental; pre-post; non-experimental); and series status (i.e., was this document part of a series of documents about the same intervention; number of documents in the series).

#### Study quality

The Jadad Scale [38,39], widely used in the medical literature, was used to assess the quality of randomized trials. On the Jadad Scale, studies are rated on a scale from 0 to 5, with the highest possible score (5) given for those with descriptions of the randomization process, an appropriate method of randomization, double-blinding (allocation concealment), an appropriate method of double-blinding, and withdrawal and dropouts.

The Newcastle-Ottawa Scale (NOS) [40].) was used to assess the quality of non-randomized studies. On the NOS, studies are rated on a scale from 0 to 9 on the basis of three main issues: study group selection, group comparability, and outcome ascertainment. NOS content validity and inter-rater reliability have been established and further evaluation is being conducted [40]. A trained research assistant and Master's student coded study quality. Inter-rater reliability (based on 16% of the eligible studies) was high, *Kappa* = 0.81. Discrepancies were resolved by consensus.

#### Feasibility

We propose 25 feasibility criteria, information about which, we suggest, is necessary to allow potential adopters to evaluate the potential feasibility of implementing interventions in their settings. We organized these criteria into the six dimensions suggested by Schoenwald and Hoagwood as important for understanding the implementation and effectiveness of empirically-based practices in real-world settings (characteristics of the intervention; practitioners; clients; service delivery context; organizational context; and service system context [24]; see Table 1 for criteria organized by dimension with examples). Schoenwald and Hoagwood suggested that evaluating the extent to which empirically-based treatments are similar to or different from frontline service practices on these dimensions could facilitate intervention developers' understanding of the transportability of empirically-based treatments. For example, the extent to which specialized training and monitoring is needed to implement an intervention effectively may impact its adoption and implementation success.

**Table 1:** Feasibility criteria, theoretical bases and coding exemplars

**Feasibility Criteria**	**Theoretical Basis**	**Coding Exemplars for “Information Included”**
**Intervention Characteristics**		
Is the treatment model of the program documented?	Compatibility/Fit	Any reference to a specific model e.g., “12-step,” “therapeutic community”
Is there a description of some or all of the services offered in the program?	Compatibility/Fit	Any description of service or program components, e.g., “the women received prenatal care and addiction services”
Is it noted that the program was documented in a manual?	Complexity	Any reference to using a “manualized” intervention or providing the name or reference for a specific manualized approach e.g., ““Nurturing Program” manual was followed”
Is there any mention of how others could adopt and implement this program?	Complexity	Any reference to other agencies adopting or implementing the program e.g., “this program has been adopted by …”
	Risk	
Is openness to local adaptation specified	Potential for adaptation	Any reference to “core features” or aspects of the program that can be changed and still maintain effectiveness e.g., “aftercare is an essential component”
Are any program implementation challenges mentioned?	Complexity	Any reference to or description of general or specific implementation challenges e.g., “the intervention experienced problems with a lack of consistency and confidentiality,” “program efforts were hampered by staff turnover and staff lack of understanding of mental health issues”
	Risk	
**Practitioner Characteristics**		
Are any staff characteristics mentioned?	Resources required	Any general or specific description of the staff involved in the intervention e.g., “all but 1 staff were female,” “staff were culturally competent,” “program was provided by a licensed clinical social worker with expertise in substance use,” “the services were provided by an obstetrician, addictions counsellor, nurse, midwife and social worker”
Is staff education level documented?		Any specific reference to the educational level required to implement the intervention or involved in the actual delivery of the program reported in the documents e.g., “degreed teachers,” “Master’s level therapists”
Is staff level of experience mentioned?		Any general or specific reference to experience e.g., “experienced drug counsellors,” “2 years of experience with this program”
Frequency or amount of any type of supervision or case/treatment discussion documented?	Resources required	Any specific reference to frequency or amount of any type of supervision or case/treatment discussion e.g., “cases were reviewed in weekly clinical staff meetings,” “full team case review occurred quarterly,” “supervision was available Monday-Friday 9-5”
Is the amount of intervention-specific staff training required for this program documented?	Resources required	Any specific reference to the training required to learn the intervention e.g., “2-day in-service,” “40-48 hours annually”
**Client Characteristics**		
Are any characteristics of the program clients described?	Compatibility/Fit	Any description of the clients/participants beyond being a women and being pregnant or parenting, including age, ethnicity, socioeconomic status, etc.
Is there a description of inclusion/exclusion criteria for the program?	Compatibility/Fit	Any reference to “inclusion,” “exclusion” or “eligibility” criteria, including more general language, e.g., “in order to be considered for the program women had to have custody of at least one child”
Are the referral sources for the program described?	Compatibility/Fit	Any description of referral sources including specific data or more general information, e.g., “the majority of participants were referred by…”
**Service Delivery Characteristics**		
Is the process of screening/assessment of potential program clients program described?	Complexity	Any description of the screening and assessment process for entry into the program, e.g., “women were assessed using a standardized battery, including.”
	Compatibility/Fit	
Is some type of planned dosage measure described?	Compatibility/Fit	Any reference to the number of planned sessions, planned length of the program, planned length of stay, e.g., “15-24 months,” “eighteen 90-minutes sessions,” “2 days per week”
	Resources required	
Is some type of actual dosage measure described?	Risk	Any reference to the number of actual sessions provided, actual length of the program or length of stay, e.g., “average number of days attended was 10,” “average length of stay was 6.3 months,” “mean number of session attended was 5”
	Resources required	
Are the program retention or withdrawal rates documented?	Risk	Any reference to “retention,” “withdrawal,” “drop-out,” or “completion/incompletion” rates e.g., “44% completed” or “27% dropped out in first 90 days”
Is the location specified?	Compatibility/Fit	Any reference to the location of the service, e.g., “hospital-based”
Is there any mention of space requirements?	Resources required	Any reference to the physical space required or involved in delivery of the intervention e.g., “14 houses and a child care centre,” “physical space on the unit designed for children,” “medical room”
Are any other client support resources described?	Resources required	Any reference to “incentives” or food, transportation or other supports e.g., “child care,” “bus tickets,” “grocery vouchers,” “$20 incentive each session,” “clothing,” “breakfast was provided”
**Organizational Characteristics**		
Is the managing agency of program specified?	Compatibility/Fit	Any reference to who “administered” or “managed” or was “responsible for” delivery of the program, e.g., university
Are the numbers of staff documented?	Resources required	Any specific reference to the number of staff required to implement the intervention or involved in the actual delivery of the program reported in the documents, e.g., “1 therapist per group, 6 used in total,” “1 director and 13 counsellors” “25 full time and 3 part time staff were involved”
**Service System Characteristics**		
Do they specify if the program is a single- or multiple-agency program?	Collaboration context	Any reference to the number of agencies involved in the intervention, e.g., “This initiative involved a collaboration between…”
Is there any mention of program cost or cost issues?	Resources required	Any general or specific references to cost or cost issues e.g., “the cost was $160 per week,” “providing transportation and housing was found to be less expensive than providing residential treatment,” “ the annual budget was $1.5 million,” “the program was funded by …”
	Risk	

Similarly, we propose that detailed information on each of these dimensions is necessary for potential adopters to evaluate the feasibility of implementing interventions in their service settings. Accordingly, our feasibility criteria are organized into Schoenwald and Hoagwood’s dimensions and are consistent with the specific variables they proposed. As well, our proposed criteria are consistent with variables identified by the SAMHSA’s National Registry of Effective and Promising Practices (NREPP) [41], as important for evaluating fit and those identified as important in understanding the acceptance and use of new practices by policy makers and service providers [42]. Initially we planned to code each document for details related to each criteria and then explore the characteristics hypothesized to impact perceptions of feasibility in combination with results from the meta-analyses of outcome studies [32-35]. Through this process we planned to explore the similarities and differences among approaches that were strong from an outcome perspective and those that were strong from a feasibility perspective. Limitations in information availability, however, led us to shift the focus to the question “Is *any* information relevant to the feasibility criteria in question included in the study documents?” In this context documents were coded based on whether or not they included information relevant to the feasibility criteria in each of the domains examined (intervention, practitioner, client, service delivery, organization, and service system characteristics). Each item was dummy coded as 1 (if the information was included) or 0 (if the information was not included). Exemplars of information considered sufficient for a rating of ‘included’ are available in Table 1. Each study was coded by one of two research assistants, who participated in coding training with the first author. In addition to the studies used for training, 20% of studies randomly selected from the set of documents were coded by both coders. Interrater reliability was high, *Kappa =* 0.80, indicating acceptable agreement between reviewers. Discrepancies were resolved by consensus with the coders and the first author Table 1.

## Results

### Document characteristics

Eleven documents could not be coded because they contained data only (*n* = 1) or the information that was provided was summarized from across multiple interventions (*n* = 10). These documents were excluded from further analyses (see Additional file 1 for a list of the included documents). The document characteristics of the remaining documents are provided in Table 2. The majority of the 121 documents were journal articles (*n* = 99) and were one in a series of documents about the same intervention (*n* = 81). Indeed, 65 unique interventions with 1–5 articles each were identified in the sample of documents examined for this study. As well, the majority of documents were predominantly outcome evaluation studies (*n* = 98) while a small proportion were intervention descriptions (*n* = 9), implementation studies (studies that examined the implementation process but did not evaluate intervention outcomes; *n* = 4), or moderator studies (*n* = 10). Given the possibility that information important to evaluating the feasibility of a particular intervention may be spread across multiple articles about the same intervention, the findings of the current study are presented by individual document (i.e., data reflect information available in each intervention document on its own; “document level”) and by intervention (i.e., data were combined across all documents about that intervention; “intervention level”).

**Table 2:** Document characteristics

**Document Characteristics**	**Full Sample of Documents (*N*= 121)**	
	***n***	**%**
**Document Type**		
Journal article	99	81.8
Book chapter	5	4.1
Dissertation	7	5.8
Report	10	8.3
**Intervention Foci**		
Pregnancy	21	17.4
Parenting	53	43.8
Both	47	38.8
**Date of Publication (years)**	Range 1978-2008	Median 1999
**Document Length (in pages)**	Range 1-250	Median 11.00
**Study Design**		
Randomized/experimental	14	11.5
Quasi-experimental	44	36.4
Pre-post	28	23.1
Non-experimental	35	29.5
**Study Series Status**		
Single publication	40	33.1
In a series	81	66.9
**Study quality**		
Jadad	Range 1-3	Mean 1.67
Newcastle-Ottawa Scale	Range 0-6	Mean 2.45

### Descriptive results

Descriptive statistics are provided in Table 2. On average, fewer than half of the feasibility details assessed were included in individual documents and approximately half could be identified if all documents related to a particular intervention were examined. Nearly all documents included some information describing the participating clients (94%) or the services offered as part of the intervention (88%). but less information was available when more specific criteria were used. For example, within the Intervention domain, while most documents included some information about the services offered as part of the intervention, only approximately half (51%) provided specific information about the treatment model and few (20%) indicated whether the intervention was documented in a manual. Notable from an adoption and implementation perspective, very few provided information about adoption and implementation of the intervention across sites (7%) or about core intervention features versus the features open to local adaptation (2%).

In the Practitioner domain, approximately two thirds of documents contained some information about the staff involved with implementing the intervention, but few (4%) documents contained information about the intervention-specific training that would be required to implement the intervention. Moreover, of the Service Delivery characteristics half to two thirds of documents provided information about planned and actual dosages of intervention and treatment retention, while far fewer (approximately 35%) provided information about clinical processes such as screening and assessment or space requirements. For each feasibility criteria examination of all materials related to a given intervention resulted in higher rates of information availability (see Table 3).

**Table 3:** Number and percentage of documents including information on feasibility criteria at Document and Intervention levels

**Feasibility Criteria**	**Document Level**		**Intervention Level**	
	**(*N*= 121)**		**(*N*= 65)**	
	***N***	**%**	***N***	**%**
**Intervention Characteristics**				
Treatment model	62	51.2	42	64.6
Description of services	107	88.4	61	93.8
Manual-based	26	21.5	17	26.2
Adoption and implementation by others	9	7.4	8	12.3
Openness to adaptation	3	2.5	3	4.6
Implementation challenges	40	33.1	29	44.6
**Practitioner Characteristics**				
Staff characteristics (any)	79	65.3	52	80.0
Education level	42	34.7	32	49.2
Experience	5	4.1	5	7.7
Supervision or case/treatment discussion	21	17.4	17	26.2
Intervention-specific staff training	5	4.1	5	7.7
**Client Characteristics**				
Client characteristics	114	94.2	65	100.0
Inclusion/exclusion criteria	58	47.9	37	56.9
Referral sources	65	53.7	44	67.7
**Service Delivery Characteristics**				
Screening and assessment process	42	34.7	30	46.2
Planned dosage	80	66.1	48	73.8
Actual dosage	65	53.7	44	67.7
Retention or withdrawal rates	72	59.5	47	72.3
Location	79	65.3	50	76.9
Space requirements	43	35.5	27	41.5
Client support resources	56	46.3	34	52.3
**Organizational Characteristics**				
Managing agency	66	54.5	36	55.4
Numbers of staff	26	21.5	21	32.3
**Service System Characteristics**				
Single- or multiple-agency	70	57.9	44	67.7
Cost or cost issues	46	38.0	32	49.2
**Total number of criteria present (out of 25**				
Range	2-23		3-24	
Mean	10.57		12.77	

### Analyses of feasibility criteria and document characteristics at the document level

We conducted *t*-tests to compare documents containing information about each feasibility criteria to those without information on three document characteristics (publication year, page length, study quality) in order to determine whether document length, publication year and study quality (where appropriate) were related to the presence of feasibility information at the document level (*n* = 121). In addition, we conducted *t*-tests comparing journal articles to all other document types combined on total number of feasibility criteria met. Lastly, we conducted bivariate correlations between total number of feasibility criteria met and the three document characteristics for journal and non-journal documents separately due to significant differences in page length (mean page length of 12 vs. 103 pages, respectively) and number of feasibility criteria met by document type.

Using a Bonferroni corrected alpha of 0.002, the *t*-tests did not reveal any significant differences in page length, publication year or study quality between documents that met each feasibility criteria and those that did not contain feasibility information. Examination of the impact of publication type (journal vs. non-journal documents) on the availability of feasibility information however revealed that non-journal documents provided information about significantly more feasibility criteria than journal articles (*t*(116) = 3.56, *p*=0.001). Correlation analyses conducted between total number of feasibility criteria met and document characteristics within each publication category, however, did not reveal any significant correlations.

For an example of how journal articles of similar scientific quality differed in their inclusion of feasibility information see Barkauskas et al., (2002; full references in Additional file 1) and Little et al., (2003). Barkauskas et al., (NOS = 5; page length = 9) provided information about 14 feasibility criteria, while Little et al., (NOS = 5; page length = 8) provided information about 5 criteria. Similarly, for an example of how non-journal documents of similar scientific quality differed see Caldwell and Zhao’s (1999) report regarding the SSTARBIRTH program (NOS = 4; page length = 86 pages) that contained information about 23 feasibility criteria and Winick and Evans’ (1997) chapter about Odyssey House (NOS = 4; page length = 17) that included information about 13 feasibility criteria or Schultz’s dissertation regarding women in the MOMS program (1997; NOS = 3; page length = 186) that contained information about 9 criteria.

### Analyses of feasibility criteria and document characteristics at the intervention level

Using intervention level data (*n* = 65) we conducted bivariate correlations between total number of feasibility criteria met and two document characteristics (number of documents in series, total page length (summed across documents)). These analyses revealed significant positive correlations between total number of feasibility criteria met across all documents related to specific interventions and number of documents in the series (*r*(65) = .61, *p*< .001) and total page length (*r*(65 = .33, *p*< .001).

## Discussion

Systematic review and analysis of 121 documents on integrated treatment programs for women with substance abuse issues and their children for the presence of information about feasibility factors revealed significant information deficits. Indeed, for over half of the feasibility criteria examined, fewer than half of the documents reviewed contained any information at all. We argue that given the importance of feasibility and its foundational concept, compatibility, in successful adoption and implementation of new practices, these deficits are likely to serve as barriers in the adoption and implementation of new interventions by service providers. These results are consistent with previous findings of deficits in client, staff, and setting-related information in intervention research in other domains such as obesity prevention [19], and mental health treatment [17,18].

Factors previously found to be associated with adoption and implementation of new practices include perceived compatibility [3,5,8,10], complexity of the new practice [5,7,8], financial and human resource requirements [5,7,8,12], and anticipated implementation challenges [5,15,19]. In order to evaluate such feasibility-related factors, service providers and decision-makers need access to information about the intervention, the practitioners required to implement the intervention, the clients for whom the intervention will be appropriate, requirements for service delivery, organizational demands, and service system characteristics [2,24]. Notably, the majority of documents in this systematic review did not include information about the program costs or material resources required for implementing the studied intervention. Moreover, the majority of documents did not provide any detailed information (e.g., education, training, experience) about the staff who provided the studied treatment or who would be required to implement the intervention in a real-world setting. Even regarding the intervention itself, information was scant on some variables, such as whether or not the intervention was manualized. In contrast, most documents provided at least some information about client characteristics and described some aspects of the interventions. When all documents about a particular intervention were combined in order to examine feasibility factors, the availability of information improved. Notably, at the intervention level, the amount of feasibility information available was related to the number of documents about the intervention and the total page length of documents available. It is not clear, however, that having feasibility information spread across multiple documents and dozens of pages of information is helpful to service providers or agency decision-makers.

Possible explanations for the general paucity of feasibility-related criteria explored in this study include a historical lack of attention to these issues thereby masking an improved current state of the literature, the overall quality of research and reporting within a particular domain of research, and space limitations. Examination of the provision of information about feasibility factors in relation to publication year revealed that the availability of information was not related to publication year, suggesting that the lack of feasibility information is both a historical and current issue. Admittedly, focused attention on the research practice gap and efforts to close it are relatively recent (year 2000). The goal of this study is not to criticize the authors of the reviewed documents but to explore a relatively unexamined factor (feasibility) that may have contributed to and may be continuing to contribute to the research-practice gap. Notably, the current study also found no relation between study quality and any of the feasibility factors. In the present study, as with the substance abuse treatment field generally, most intervention studies did not report high quality designs [43]. Studies included in the meta-analyses were assessed as being of low to moderate quality[32-35], although it was unclear if the scores reflected study quality per se or the reporting of study quality elements. Higher quality studies with better reporting in the domain of integrated treatment services for women with substance abuse issues who are pregnant or parenting are recommended for future research efforts.

Regarding the possibility that information deficits found in this study could be associated with space limitations inherent in journal publications and possible editor or publisher pressure for short article lengths [19], this study found some supporting evidence. Journal articles were significantly shorter that non-journal documents and a significant positive association between page length and the overall provision of information at the intervention level was found; however, no other relations between page length and the presence of specific feasibility factors were found to be significant, so the potential impact of space limitations will require further exploration. Another aspect of the editorial and publishing process that may contribute to the noted information deficits may be the focus on reward for the scientific impact of publications with little attention to the practice and policy impacts of publications [44]. As well, the relative absence of frontline service providers and decision-makers in the intervention research planning process has been suggested as reducing the likelihood that the needs of frontline service providers are reflected in study processes and measures [3,4,24,44]. Indeed, Schoenwald and Hoagwood have argued that interdisciplinary teams are necessary given the range of expertise needed to adequately consider the six dimensions of intervention information they proposed as important and that we used in this study [24]. Other possible explanations for the paucity of information we found also exist at the level of data collection and reporting. For example, researchers may not systematically record and monitor the necessary information due to the expense associated with doing so or, alternatively, may choose not to report such information even if it is gathered [45].

As has been discussed in the context of efforts to improve the external validity and generalizability of research in medicine, health promotion, and treatment outcome studies, the implementation of reporting rules such as those used for ensuring reporting of factors affecting internal validity may promote improved information sharing [16,18,19,46,47]. Ultimately, successful diffusion of effective interventions to practice settings will likely require continued attention to internal validity (the extent to which observed outcomes can be attributed to the intervention), continued attention to external validity (the extent to which observed intervention outcomes can be generalized to other settings (e.g., real world settings)) [16], and increased attention to feasibility (the extent to which the intervention can be successfully carried out or implemented in a given setting). Accordingly, in addition to existing reporting standards for internal validity and external validity, it is proposed that a set of feasibility reporting criteria, such as those examined in this study, be considered by intervention developers, researchers and editorial boards to evaluate the presence of the information necessary to evaluate feasibility for real-world settings. Moreover, future work should focus on identifying the key pieces of information required at different stages of the implementation process and how to best make such information available to those who make intervention implementation decisions.

This review is limited in that it focused specifically on studies of integrated treatment programs for women with substance abuse issues and their children that met the specified eligibility criteria, although the findings are consistent with other similar reviews in other areas [21-23]. As well, despite a comprehensive grey literature search, the majority of documents included were journal articles and important sources of information for intervention implementation decision-making may have been missed. As well, the work of this study is exploratory and the coding scheme is preliminary. It may be the case that important feasibility-related items were not included or that use of different items would have resulted in different findings. Indeed, the validity of our criteria as measures of the underlying construct of feasibility may be limited thereby limiting their connection to the actual process of adoption and implementation. Ultimately future work is needed across different intervention domains to establish a strong standardized feasibility coding system that can be meaningfully integrated into systems for providing evaluations of interventions that extend beyond treatment efficacy. We see this review as a first step in that process.

Accordingly, it is recommended that future steps include the following: 1) research be conducted regarding service provider and decision-maker perspectives on the relative importance of various feasibility factors and the criteria proposed in the present study; 2) continued development and evaluation of feasibility criteria with the goal of providing service providers and decision-makers with a standardized tool to contribute to intervention decision-making; and 3) that intervention developers collaborate with frontline service providers and decision-makers from the outset of intervention development and evaluation to ensure that research protocols address feasibility issues in study implementation and documentation processes.

## Conclusions

The results of this study indicate that feasibility-related information important in intervention adoption and implementation decisions by frontline services is lacking in the both journal and non-journal documents regarding treatments for women with substance use issues and their children. We suggest that this paucity of information may be contributing to the persistence of the research-practice gap in treatment for women with substance abuse issues and their children. Of course, documents in general, and journal articles in particular, are not the main vehicles of effective diffusion of treatment innovations and they serve multiple purposes beyond dissemination of information for implementation. Nevertheless they continue to serve as one of the primary sources of information for best practice literature reviews and clinical practice guidelines (e.g., [48]), and journal articles in particular continue to be used as critical criterion for achieving “empirically-supported” status (e.g., [49]). The paucity of information important to feasibility evaluation, as well as our continued need to better understand decisions to adopt new interventions, suggests an opportunity for journal article authors and editors to consider the reporting of feasibility factors in future publications. Moreover, this study recommends specific feasibility criteria organized into six dimensions (intervention, practitioner, client, service delivery, organizational, and service systems characteristics) that have been suggested as important for understanding the transportability of new practice and highlight the need for interdisciplinary collaboration [24].

To our knowledge, this study is the first systematic review and analysis of studies evaluating the impact of integrated treatment programs for the presence of feasibility information. Given that approximately one third of people with drug dependence are women of child-bearing age [27], substance use during pregnancy is a major public health concern [25], and burden of suffering due to maternal substance abuse is great, the findings from this study are noteworthy and support the need for better reporting on integrated treatment programs for women with substance abuse issues and their children.

## Competing interests

The authors declare that they have no competing interests.

## Authors’ contributions

AN, KM, WS, and LT conceived of the program of research, while JH conceived of this particular study. JH developed the coding system, supervised coding, conducted analyses and drafted the manuscript. AN helped develop the coding system and draft the manuscript. AS helped code data and helped with analyses. SR coded data, contributed to analyses, and helped draft the manuscript. All authors read and approved the final manuscript.

## Supplementary Material

Additional File 1 References for study documents (N=121). Click here for file
