# Supplementary material for: Reporting of feasibility factors in publications on integrated treatment programs for women with substance abuse issues and their children: a systematic review and analysis
Source: Health Res Policy Syst. 2012 Dec 7;10:37. doi: 10.1186/1478-4505-10-37 (PMC3547724; doi:10.1186/1478-4505-10-37)
Supplement: Additional File 1 — References for study documents (N=121). [file 1478-4505-10-37-S1.docx]

**Appendix B Study documents (N=121)**

| Armstrong MA, Gonzales V, Lieberman L, Carpenter DM, Pantoja PM, Escobar GJ: **Perinatal substance abuse intervention in obstetric clinics decreases adverse neonatal outcomes.** *J Perinatol* 2003, **23:**3-9. |
| --- |
| Armstrong MA, Lieberman L, Carpenter DM, Gonzales V, Usatin MS, Newman L. et al.: **Early Start: an obstetric clinic-based perinatal substance abuse intervention program**. *Qual Manag Health Care* 2001, **9**:6-15. |
| Barkauskas VH, Low LK, Pimlott S: **Health outcomes of incarcerated pregnant women and their infants in a community-based program.** *J Midwifery Womens Health* 2002, **47:**371-379. |
| Belcher HME, Butz AM, Wallace P, Hoon AH, Reinhardt E, Reeves SA et al.: **Spectrum of early intervention services for children with intrauterine drug exposure.** *Infants Young Child* 2005, **18**:2-15. |
| Berkowitz G, Brindis CD, Clayson Z, Peterson S: **Options for Recovery: promoting success among women mandated to treatment.** *J Psychoactive Drugs* 1996, **28**:31-38. |
| Berkowitz G, Brindis C, Peterson S: **Substance use and social outcomes among participants in perinatal alcohol and drug treatment.** *Womens Health: Res Gend Behav Policy* 1998, **4**:231-254. |
| Besinger B. **Mothers in addiction treatment: the role of onsite childcare.** *PhD thesis.* University of Cincinnati, Department of Psychology; 2003. |
| Brindis CD, Berkowitz G, Clayson Z: **Options for Recovery: promoting perinatal drug and alcohol recovery, child health, and family stability.** *J Drug Issues* 1997, **27:**607-624. |
| Brindis CD, Clayson Z, Berkowitz G. (1997): **Options for Recovery: California's perinatal projects.** *J Psychoactive Drugs* 1997, **29**:89-99. |
| Brown VB, Melchior LA, Waite-O'Brien N, Huba GJ. **Effects of women-sensitive, long-term residential treatment on psychological functioning of diverse populations of women.** *J Subst Abuse Treat* 2002, **23**:133-144. |
| Caldwell D, Zhao AR: *SSTARBIRTH Final Program Evaluation Report.* Rhode Island; 1999. |
| Camp JM, Finkelstein N: **Parenting training for women in residential substance abuse treatment: results of a demonstration project.** *J Subst Abuse Treat* 1997, **14:**411-422. |
| Carroll KM, Chang G, Behr HM, Clinton B, Kosten TR: **Improving treatment outcome in pregnant, methadone-maintained women.** *Am J Addict* 1995, **4**:56-59. |
| Chang G, Carroll K, Behr HM, Kosten TR: **Improving treatment outcome in pregnant opiate-dependent women.** *J Subst Abuse Treat* 1995, **9:**327-330. |
| Colenso HM: **Psychosocial correlates of drop out in a substance abuse treatment program for mothers.** *PhD thesis.* New York University, School of Social Work; 2002. |
| Coletti SD, Hughes PH, Landress HJ, Neri RL, Sicilian DM, Williams KM, et al.: (1992). **PAR Village: Specialized intervention for cocaine abusing women and their children.** *J Fla Med Assoc* 1992, **79**:701-705. |
| Coletti SD, Schinka JA, Hughes PH, Hamilton NL, Neri RL: S**pecialized therapeutic community treatment for chemically dependent women and their children.** In *Community as Method: Therapeutic Communities for Special Populations and Special Settings.* Edited by DeLeon G. Westport, Connecticut: Praeger Publishers; 1997:117-128. |
| Coletti SD, Schinka JA, Hughes PH, Hamilton NL, Renard CG, Sicilian DM, Urmann CF, Neri RL: **PAR village for chemically dependent women: philosophy and program elements.** *J Subst Abuse Treat* 1995, **12**:289–296. |
| Comfort M, Kaltenbach KA: **Biopsychosocial characteristics and treatment outcomes of pregnant cocaine-dependent women in residential and outpatient substance abuse treatment.** *J Psychoactive Drugs* 1999, **31:**279-289. |
| Comfort M, Sockloff A, Loverro J, Kaltenbach KA: **Multiple predictors of substance-abusing women's treatment and life outcomes: A prospective longitudinal study.** *Addict Behav* 2003, **28**:199-224. |
| Conners NA, Bokony P, Whiteside-Mansell L, Bradley RH, Liu J: **Addressing the treatment needs of children affected by maternal addiction: challenges and solutions.** *Eval Program Plann* 2004, **27**:241-247. |
| Conners NA, Bradley RH, Whiteside-Mansell L, Crone, CC: **A comprehensive substance abuse treatment program for women and their children: an initial evaluation.** *J Subst Abuse Treat* 2001, **21**:67-75. |
| Conners NA, Grant A, Crone CC, Whiteside-Mansell L: **Substance abuse treatment for mothers: treatment outcomes and the impact of length of stay.** *J Subst Abuse Treat* 2006, **31**:447-456. |
| Corse SJ, Smith M: **Reducing substance abuse during pregnancy: discriminating among levels of response in a prenatal setting.** *J Subst Abuse Treat* 1998, **15**:457-467. |
| Elk R, Mangus L, LaSoya RJ, Rhoades H, Andres R, Grabowski J: **Behavioral interventions: effective and adaptable for the treatment of pregnant, cocaine-dependent women.** *J Drug Issues* 1997, **27**:625-658. |
| Elk R, Schmitz J, Spiga R, Rhoades H, Andres R, Grabowski J: **Behavioral treatment of cocaine-dependent pregnant women and TB-exposed patients.** *Addict Behav* 1995, **20:**533-542. |
| Ellwood DA, Sutherland P, Kent C, O'Connor M: **Maternal narcotic addiction: pregnancy outcome in patients managed by a specialized drug-dependency antenatal clinic.** *Aust N Z J Obstet Gynaecol* 1987, **27**:92-98. |
| Evenson RC, Binner PR, Cho DW, Schicht WW, Topolski JM: **An outcome study of Missouri's CSTAR alcohol and drug abuse programs.** *J Subst Abuse Treat* 1998*,* **15**:143-150. |
| Farrow JA, Watts H, Krohn MA, Olson HC: **Pregnant adolescents in chemical dependency treatment: description and outcomes.** *J Subst Abuse Treat* 1999, **16**:157-161. |
| Field TM, Scafidi F, Pickens J, Prodromidis M, Pelaez-Nogueras M, Torquati J, et al.: **Polydrug-using adolescent mothers and their infants receiving early intervention.** *Adolescence* 1998, **33**:117-143. |
| Graham AV, Graham NR, Sowell A, Ziegler H: **Miracle Village: A recovery community for addicted women and their children in public housing.** *J Subst Abuse Treat* 1997, **14**:275-284. |
| Gwadz MV, Leonard NR, Cleland CM, Riedel M, Arredondo GN, Wolfe H, et al.: **Behavioral interventions for HIV infected and uninfected mothers with problem drinking.** *Addict Res Theory* 2008, **16**:47-65. |
| Haller DL, Knisely JS, Elswick RK, Dawson KS, Schnoll SH: **Perinatal substance abusers: factors influencing treatment retention.** *J Subst Abuse Treat* 1997, **14**:513-519. |
| Haller DL, Miles DR, Dawson KS: **Psychopathology influences treatment retention among drug-dependent women.** *J Subst Abuse Treat* 2002, **23**:431-436. |
| Harshman WL: **A comparison of the effects of a gender-specific and traditional model of substance abuse treatment within the therapeutic community on treatment success.** *PhD thesis.* Wayne State University; 1999. |
| Higgins PG, Clough DH, Wallerstedt C: **Drug-taking behaviours of pregnant substance abusers in treatment.** *J Adv Nurs* 1995, **22**:425-432. |
| Hogan TMS, Myers BJ, Elswick RK: **Child abuse potential among mothers of substance-exposed and nonexposed infants and toddlers.** *Child Abuse Negl* 2006, **30:**145-156. |
| Hood P, Knight DK, Logan SM: *Mutually beneficial collaboration: Using evaluation to improve service delivery. Lessons learned: Residential substance abuse treatment for women and their children* (DHHS Publication No. SMA 03-3787). Rockville, MD: Center for Substance Abuse Treatment; 1999. |
| Hughes PH, Coletti SD, Neri RL, Urmann CF: **Retaining cocaine-abusing women in a therapeutic community: the effect of a child live-in program.** *Am J Public Health* 1995, **85**:1149-1152. |
| Ingersoll KS, Knisely JS, Dawson KS, Schnoll SH: **Psychopathology and treatment outcome of drug dependent women in a perinatal program.** *Addict Behav* 2004, **29:**731-741. |
| Jansson LM, Svikis D, Lee J, Paluzzi P: **Pregnancy and addiction: a comprehensive care model.** *J Subst Abuse Treat* 1996, **13**:321-329. |
| Jansson LM, Velez M. **Understanding and treating substance abusers and their infants.** *Infants Young Child* 1999;**11**:79–89. |
| Kern JK, West EY, Grannemann BD, Greer TL, Snell LM, Cline LL et al.: **Reductions in stress and depressive symptoms in mothers of substance-exposed infants participating in a psychosocial program.** *Matern Child Health J* 2004, **8**:127-136. |
| Killeen T, Brady KT: **Parental stress and child behavioral outcomes following substance abuse residential treatment: follow-up at 6 and 12 months.** *J Subst Abuse Treat* 2000, **19**:23-29. |
| Knight DK, Wallace GL: **Where are the children? An examination of children's living arrangements when mothers enter residential drug treatment.** *J Drug Issues* 2003, **33**:305-324. |
| Knight DK, Logan SM, Simpson DD: **Predictors of program completion for women in residential substance abuse treatment.** *Am J Drug Alcohol Abuse* 2001, **27**:1-18. |
| Knight DK, Wallace GL, Joe GW, Logan SM: **Change in psychosocial functioning and social relations among women in residential substance abuse treatment.** *J Subst Abuse* 2001, **13**:533-547. |
| Knight DK, Hood PE, Logan SM, Chatham LR: **Residential treatment for women with dependent children: one agency’s approach*.*** *J Psychoactive Drugs* 1999, **31:**339-351. |
| Koren G: **Breaking the Cycle: reflections on the first nine years.** *J FAS Int* 2004*,* **2**:e2. |
| Kubiak SP, Young A, Siefert K, Stewart A: **Pregnant, substance-abusing, and incarcerated: exploratory study of a comprehensive approach to treatment.** *Fam Soc* 2004, **85:**177-186. |
| Kukko H, Halmesmaki E: **Prenatal care and counseling of female drug-abusers: effects on drug abuse and perinatal outcome.** *Acta Obstet Gynecol Scand* 1999, **78:**22-26. |
| Kyei-Aboagye K, Acker DB, MacBain D: **The effect of postdetoxification drug-free residential living on birth outcome in the pregnant drug abuser.** *Subst Abus* 1998, **19**:123-128. |
| La France SV, Mitchell J, Damus K, Driver C, Roman G, Graham E, et al.: **Community-based services for pregnant substance-using women.** *Am J Public Health* 1994, **84**:1688-1689. |
| Lafazia MA (Ed): *Washington State MOMS Project: Perinatal Research and Demonstration Project.* Washington: Department of Social and Health Services, Division of Alcohol and Substance Abuse; 1999. |
| Laken MP, McComish JF, Ager J: **Predictors of prenatal substance use and birth weight during outpatient treatment.** *J Subst Abuse Treat* 1997, **14**:359-366. |
| Leslie M: **Evaluation of Breaking the Cycle (1995-1997).** *IMPrint* 1998, **22**:8-12. |
| Little BB, Snell LM, VanBeveren TT, Crowell RB, Trayler S, Johnston WL: **Treatment of substance abuse during pregnancy and infant outcome.** *Am J Perinatol* 2003, **20**:255-262. |
| Luthar SS, Suchman NE: **Relational psychotherapy mothers' group: a developmentally informed intervention for at-risk mothers.** *Dev Psychopathol* 2000, **12:**235-253. |
| Luthar S, Suchman NE, Altomare M: **Relational psychotherapy mother's group: a randomized clinical trial for substance abusing mothers.** *Dev Psychopathol* 2007, **19**:243-261. |
| Marsh JC: **Women helping women: the evaluation of an all female methadone maintenance program in Detroit.** In *Drug Dependence and Alcoholism, Volume 1: Biomedical Issues* Edited by Schecter AJ. New York: Plenum Press; 1981:893-900. |
| Marsh JC, D'Aunno TA, Smith BD: **Increasing access and providing social services to improve drug abuse treatment for women with children.** *Addiction* 2000, **95**:1237 - 1247. |
| McCarthy JJ, Leamon MH, Parr MS, Anania B: **High-dose methadone maintenance in pregnancy: Maternal and neonatal outcomes.** *Am J Obstet Gynecol* 2005, **193**:606-610. |
| McComish JF, Greenberg R, Ager J, Chruscial H, Laken MA: **Survival analysis of three treatment modalities in a residential substance abuse program for women and children.** *Outcomes Manag Nurs Pract* 2000, **4**:71-77. |
| McComish JF, Greenberg R, Ager J, Essenmacher L, Orgain LS, Bacik WJ: **Family-focused substance abuse treatment: a program evaluation.** *J Psychoactive Drugs* 2003, **35**:321-331. |
| McComish JF, Greenberg R, Kent-Bryant J, Chruscial H, Ager J, Hines F, et al.: **Evaluation of a grief group for women in residential substance abuse treatment.** *Subst Abus* 1999, **20:**45-58. |
| McKay JR, Gutman M, McLellan TA, Lynch KG, Ketterlinus R: **Treatment services received in the Casaworks for families program.** *Eval Rev* 2003, **27**:629-655. |
| McLellan TA, Gutman M, Lynch K, McKay JR, Ketterlinus R, Morgenstern J, et al.: **One-year outcomes from the Casaworks for families intervention for substance-abusing women on welfare.** *Eval Rev* 2003, **27**:656-680. |
| McMurtie C, Rosenberg KD: *PACE: Final report and evaluation.* New York: 1994. |
| McMurtrie C, Rosenberg KD, Kerker BD, Kan J, Graham EH: **A unique drug treatment program for pregnant and postpartum substance-using women in New York City: results of a pilot project: 1990-1995.** *Am J Drug Alcohol Abuse* 1995, **25**:701-703. |
| Melchior LA, Huba GJ, Brown VB: *Evaluation of the Center for Substance Abuse Treatment grant: PROTOTYPES moms & kids drug treatment-prevention project October 1, 1992 – September 30, 1997.* Culver City, CA: The Measurement Group; 1997. |
| Melchior LA, Huba GJ, Brown VB: *Evaluation of the residential treatment program for women and their children: PROTOTYPES women’s center.* Culver City, CA: The Measurement Group; 1999. |
| Metsch LR, Wolfe HP, Fewell R, McCoy CB, Elwood WN, Wohler-Torres B, et al.: **Treating substance-using women and their children in public housing: preliminary evaluation findings.** *Child Welfare J* 2001, **80**:199-220. |
| Moore J, Finkelstein N: **Parenting services for families affected by substance abuse.** *Child Welfare* 2001, **80:**221-238. |
| Morgenstern J, Nakashian M, Woolis DD, Gibson FM, Bloom NL, Kaulback BG: **Casaworks for families: a new treatment model for substance-abusing parenting women on welfare.** *Eval Rev* 2003, **27**:583-596. |
| Mosley TM: **PROTOTYPES: an urban model program of treatment and recovery services for dually diagnosed perinatal program participants.** *J Psychoactive Drugs* 1996, **28**:381-388. |
| Motomura M. *Caring Connections Final Evaluation Report.* Windsor, ON; 2006. |
| Motz M, Leslie M, Pepler DJ, Moore TE, Freeman PA : *Breaking the Cycle: Measures of Progress 1995 - 2005.* Toronto; 2006 |
| Myers BJ, Dawson KS, Britt GC, Lodder DE, Meloy LD, Saunders MK, et al.: **Prenatal cocaine exposure and infant performance on the Brazelton Neonatal Behavioral Assessment Scale.** *Subst Use Misuse* 2003, **38:**2065-2096. |
| Nardi DA: **Parent-infant interaction during perinatal addiction treatment.** *Issues Compr Pediatr Nurs* 1994, **17:**161-175. |
| Nardi DA: **Parenting during recovery: an analysis of parenting during the first year in a chemical dependency treatment program.** *PhD thesis.* Loyola University of Chicago; 1994. |
| Nardi DA: **Risk factors, attendance, and abstinence patterns of low-income women in perinatal addiction treatment: lessons from a 5-year program.** *Issues Ment Health Nurs* 1997, **18**:125-138. |
| Niccols A, Sword W: **"New Choices" for substance-using mothers and their children: preliminary evaluation.** *J Subst Use* 2005, **10**:239-251. |
| Nishimoto RH, Roberts AC: **Coercion and drug treatment for postpartum women.** *Am J Drug Alcohol Abuse* 2001, **27**:161-181. |
| Noether CD, Brown V, Finkelstein N, VanDeMark NR, Marris LS, Graeber C: **Promoting resiliency in children of mothers with co-occuring disorders and histories of trauma: impact of a skills-based intervention program on child outcomes.** *J Community Psychol* 2007, **35**:823-843. |
| Ortega S, Salmon K, Barry M, Ampy L: *Lessons Learned: Serving Pregnant and Parenting Women in Substance Abuse Treatment Programs.* Florida: Florida Department of Children and Families; 2002. |
| Pepler DJ, Moore TE, Motz M, Leslie M : *Breaking the Cycle: The Evaluation Report (1995 - 2000).* Toronto: Breaking the Cycle; 2002. |
| Porowski AW, Burgdorf K, Herrell JM: **Effectiveness and sustainability of residential substance abuse treatment programs for pregnant and parenting women.** *Eval Program Plann* 2004, **27**:191-198. |
| Sacks JY, Sacks S, Harle M, De Leon G: H**omelessness prevention therapeutic community for addicted mothers.** *Alcohol Treat Q* 1999, **17**:33-51. |
| Sacks S, Sacks JY, McKendrick K, Pearson FS, Banks S, Harle M: **Outcomes from a therapeutic community for homeless addicted mothers and their children**. *Adm Policy Ment Health* 2004, **31**:313-338. |
| Saunders E: **Project Together: Serving substance-abusing mothers and their children in Des Moines.** *Am J Public Health* 1992, **82**:1166-1167. |
| Saunders E J: **A new model of residential care for substance-abusing women and their children.** *Adult Resid Care J* 1993, **7**:104-117. |
| Schinka JA, Hughes PH, Coletti SD, Hamilton NL, Renard CG, Urmann CF, et al.: **Changes in personality characteristics in women treated in a therapeutic community.** *J Subst Abuse Treat* 1999, **16**:137-142. |
| Schretzman MK: **Voices of successful women: graduates of a residential treatment program for homeless addicted women with their children.** *PhD thesis.* City University of New York; 1999. |
| Schulz L: **Concomitants of success in a perinatal substance abuse treatment program.** *PhD thesis.* George Mason University; 1997. |
| Schumacher JE, Siegal SH, Socol JC, Harkless S, Freeman K: **Making evaluation work in a substance abuse treatment program for women with children: Olivia's House.** *J Psychoactive Drugs* 1996, **28**:73-83. |
| Scully M, Geoghegan N, Corcoran P, Tiernan M, Keena E: **Specialized drug liaison midwife services for pregnant opioid dependent women in Dublin, Ireland.** *J Subst Abuse Treat* 2004, **26**:27-33. |
| Siefert K, Pimlott S: **Improving pregnancy outcome during imprisonment: a model residential care program.** *Soc Work* 2001, **46**:125-134. |
| Simpson KD: **Mothers in recovery: women's perceptions of a parenting program in an outpatient addiction treatment centre.** *PhD thesis.* University of Windsor; 2004. |
| Sowers KM, Ellis RA, Washington TA, Currant M: **Optimizing treatment effects for substance-abusing women with children: an evaluation of the Susan B. Anthony Center.** *Res Soc Work Pract* 2002, **12**:143-158. |
| Stevens SJ, Arbiter N: **A therapeutic community for substance-abusing pregnant women and women with children: process and outcome.** *J Psychoactive Drugs* 1995, **27**:49-56. |
| Stevens SJ, Arbiter N, Glider P: **Women residents: Expanding their role to increase treatment effectiveness.** *Int J Addict* 1989, **24**:425-434. |
| Stevens SJ, Arbiter N, McGrath R: **Women and children: therapeutic community substance abuse treatment.** In *Community as Method: Therapeutic Communities for Special Populations and Special Settings* Edited by De Leon G. Westport, Connecticut: Praeger Publishers; 1997:129-141. |
| Stevens SJ, Patton T: **Residential treatment for drug addicted women and their children: effective treatment strategies.** *Drugs Soc* 1998, **13**:235-249. |
| Strantz IH, Welch SP: **Postpartum women in outpatient drug abuse treatment: correlates of retention/completion.** *J Psychoactive Drugs* 1995, **27**:357-373. |
| Suchman N, Mayes L, Conti J, Slade A, Rounsaville B: **Rethinking parenting interventions for drug-dependent mothers: from behavior management to fostering emotional bonds.** *J Subst Abuse Treat* 2004, **27**:179-185. |
| Sweeney PJ, Schwartz RM, Mattis NG, Vohr B: **The effect of integrating substance abuse treatment with prenatal care on birth outcome.** *J Perinatol* 2000, **20:**219-224. |
| Sword W, Niccols A, Fan A: **"New Choices" for women with addictions: perceptions of program participants.** *BMC Public Health* 2004, **4:**10. |
| Taillac C, Goler N, Armstrong M, Haley K, Osejo V: **Early Start: an integrated model of substance abuse intervention for pregnant women.** *Perm J* 2007, **11**:5–11. |
| Tanney MR, Lowenstein V: **One-stop shopping: description of a model program to provide primary care to substance-abusing women and their children.** *J Pediatr Health Care,* 1997, **11**:20-25. |
| Toussaint DW, VanDeMark NR, Bornemann A, Graeber C: **Modifications to the trauma recovery and empowerment model (TREM) for substance-abusing women with histories of violence: Outcomes and lessons learned at a Colorado substance abuse treatment center.** *J Community Psychol* 2007, **35**:879-894. |
| Velez ML, Jansson LM, Montoya ID, Schweitzer W, Golden A, Svikis D: **Parenting knowledge among substance abusing women in treatment.** *J Subst Abuse Treat* 2004, **27**:215-222. |
| Velez M, Peirce J, Svikis D, Walters V, Jansson L: **Clinical treatment-based research: Assessment and intervention for violence in pregnant women attending substance abuse treatment.** *NIDA Res Monogr* 2002, **183**:112-115. |
| Volpicelli JR, Markman I, Monterosso J, Filing J, O'Brien CP: **Psychosocially enhanced treatment for cocaine-dependent mothers: evidence of efficacy.** *J Subst Abuse Treat* 2000, **18**:41-49. |
| Weisdorf T, Parran TV, Graham A, Snyder C: **Comparison of pregnancy-specific interventions to a traditional treatment program for cocaine-addicted pregnant women.** *J Subst Abuse Treat* 1999, **16**:39-45. |
| West M, Frankel B, Dalton J: **Treatment progress of substance dependent mothers and their children at Family House.** In *Drug Dependence and Alcoholism Volume 1: Biomedical Issues.* Edited by Schecter AJ. New York: Plenum Press; 1981:789-799. |
| Wexler HK, Cuadrado M, Stevens SJ: **Residential treatment for women: behavioral and psychological outcomes.** *Drugs Soc* 1998, **13**:213-233. |
| Whiteside-Mansell L, Crone CC, Conners NA: **The development and evaluation of an alcohol and drug prevention and treatment program for women and children.** *J Subst Abuse Treat* 1999, **16**:265-275. |
| Winick C, Evans JT: **A therapeutic community program for mothers and their children.** In *Community as Method: Therapeutic Communities for Special Populations and Special Settings.* Edited by: DeLeon G. Westport, Connecticut: Praeger Publishers; 1997:143-159. |
| Wobie K, Eyler FD, Conlon M, Clarke L, Behnke M: **Women and children in residential treatment: Outcomes for mothers and their infants.** *J Drug Issues* 1997, **27**:585-606. |
| Worley L, Conners N, Crone C, Williams V, Bokony P: **Building a residential treatment program for dually diagnosed women with their children.** *Arch Womens Ment Health* 2005, **8**:105 -111. |
| Zlotnick C, Franchino K, St.Claire N, Cox K, St.John M: **The impact of outpatient drug services on abstinence among pregnant and parenting women.** *J Subst Abuse Treat* 1996, **13**:195-202. |
